# The effectiveness of vasodilators on chronic obstructive pulmonary disease: A systematic review and meta-analysis

**DOI:** 10.1097/MD.0000000000039794

**Published:** 2024-11-15

**Authors:** Ningxin Han, Hui Qi, Yujie Yin, Yi Liu, Peipei Jin, Yunlong Hou, Zhenhua Jia

**Affiliations:** a Graduate School, Hebei Medical University, Shijiazhuang, China; b Hebei Academy of Integrated Traditional Chinese and Western Medicine, Shijiazhuang, China; c State Key Laboratory for Innovation and Transformation of Luobing Theory, Shijiazhuang, China; d Graduate School, Hebei University of Traditional Chinese Medicine, Shijiazhuang, China; e Hebei Yiling Hospital, High-level TCM Key Disciplines of National Administration of Traditional Chinese Medicine – Luobing Theory, Shijiazhuang, China.

**Keywords:** chronic obstructive pulmonary disease, meta-analysis, pulmonary capillaries, vasodilator

## Abstract

**Background:**

Chronic obstructive pulmonary disease (COPD) is a complex progressive disease. Some vasodilators have been reported with therapeutic potential to protect vascular function therefore may delay the progression of COPD.

**Methods:**

We searched PubMed, Embase, Cochrane Library, Web of Science, OVID and Clinicaltrials.gov database for eligible randomized controlled trials (RCTs) published before January 1, 2024. RCTs which treatment with vasodilators to COPD patients were included. Gas-blood exchange indicators were the primary outcomes, and ventilation function and quality of life indicators were the secondary outcomes. Mean differences with 95% confidence intervals were extracted. Subgroup analysis of vasodilator category and COPD complicated with or without pulmonary hypertension (PH) were performed. The risk of bias was assessed using Cochrane risk of bias tool, and the meta-analysis was conducted.

**Results:**

Twenty studies with a total sample size of 986 were included. The results showed that the 2 types of drugs in vasodilators included PDE-5 inhibitors could improve DLCO (MD = 6.56 [95% CI (1.74, 11.39)], *P* = .008) and iNO could reduce PaCO_2_ (MD = −0.10 [95% CI (−0.17, −0.03)], *P* = .006). Vasodilators could reduce PaCO_2_ in COPD complicated with PH (COPD-PH) (MD = −0.10 [95% CI (−0.17, −0.03)], *P* = .006). There were no statistically significant differences in FEV1 (MD = 0.02 [95% CI (−0.11, 0.16)], *P* = .74), FEV1% predicted (MD = 0.07 [95% CI (−1.90, 2.05)], *P* = .94), FEV1/FVC (MD = 0.70 [95% CI (−4.02, 5.42)], *P* = .77) and *V*_*E*_*/V*_*CO2*_ (MD = −0.17 [95% CI (−2.39, 2.05)], *P* = .88) levels. The total SGRQ score was significantly lower in vasodilator groups (MD = −5.53 [95% CI (−9.81, −1.24)], *P* = .01).

**Conclusions:**

The therapeutic effects of vasodilators for COPD are controversial. In this meta-analysis, vasodilators have benefits in improving gas-blood exchange function and quality of life in COPD patients. However, vasodilators may have a limited capacity to improve pulmonary function.

## 1. Introduction

Chronic obstructive pulmonary disease (COPD), is the most prevalent disease (approximately 11.7%) and the fourth leading cause of death globally.^[1]^ COPD is characterized by progressive airflow limitation and chronic inflammation in the airways, lung parenchyma and pulmonary vascular system.^[2]^ The current pharmacological treatment of COPD is mainly based on bronchodilators, which can only relieve symptoms of airway obstruction with little or no effect on disease progression.^[3]^ Therefore, there is an urgent need to explore the pathogenesis of COPD and find new therapeutic targets for preventing the progression of COPD. Multiple large cohort studies^[4–6]^ have shown that the use of vasodilators such as angiotensin-converting enzyme inhibitors (ACEIs) and angiotensin II receptor blockers (ARBs) can prevent smoking-induced rapid declines in lung function and are associated with slower progression of emphysema. Rapid decline in lung function due to smoking is associated with slower progression of emphysema, which may help to slow the progression of COPD. Thus, vasodilators may be potential interventional drugs for COPD treatment.

Cigarette smoke is the most important and extensively studied risk factor for COPD. It has been proven that harmful substances in cigarette smoke not only damage the airway epithelium and vascular endothelium but also have adverse effects on the microvascular system of multiple organs, such as the heart, brain, and kidney,^[7]^ resulting in reduced blood flow and damage to organs. Studies have proved that drugs such as renin-angiotensin system blockers, aspirin and anthocyanins can protect the function of these organs by improving microvascular blood flow.^[8–10]^ A MESA COPD study showed that pulmonary microvascular blood flow (PMBF) was reduced in the lungs of patients with mild COPD, including areas without overt emphysema. Notably, the reduction in blood flow occurred throughout the progression of COPD. Several clinical studies have demonstrated that drugs with vasodilatory effects, such as phosphodiesterase 5 (PDE-5) inhibitors and ACEIs, could increase PMBF and delay COPD progression, suggesting the therapeutic potential of vasodilators for early-stage COPD.^[11,12]^

Vasodilators such as ACEIs, ARBs, calcium antagonists (CCBs), and nitrates are commonly used in cardiovascular diseases and have good therapeutic effects; PDE-5 inhibitors, nitric oxide (NO), prostacyclins, and endothelin receptor antagonists are commonly used in patients with pulmonary arterial hypertension (PH); however, the effects of these drugs on ventilation and expiratory function in COPD are not yet known. Therefore, we intend to systematically evaluate the therapeutic effects and potential clinical value of vasodilators for patients with COPD based on a meta-analysis. Given that different vasodilators have different therapeutic targets and effects, in this study, we classified the commonly used vasodilators into 8 groups, including ACEIs, ARBs, CCBs, nitrates, PDE-5 inhibitors, NO, prostacyclins and endothelin receptor antagonists.

## 2. Methods

### 2.1. Protocol and registration

This systematic review and meta-analysis adhered to the preferred reporting items for systematic reviews and meta-analyses (PRISMA) statement.^[13]^ The protocol was registered in the International prospective register of systematic reviews (PROSPERO, CRD42022300332).

### 2.2. Search strategy

The MEDLINE, Cochrane Central Register of Controlled Trials, Embase, Web of Science and Ovid databases were searched for potentially eligible published studies. Articles published before January 1, 2024. To prevent potential publication bias, trial registries (https://www.clinicaltrials.gov) were screened for ongoing and completed trials. In addition, relevant references were added by the snowball approach to retrieve additional relevant studies. The retrieval strategy was mainly composed of MeSH subject words and free words with the following terms: “COPD,” “ACEIs,” “ARBs,” “CCBs,” “nitrates,” “prostacyclin,” “NO,” “PDE-5 inhibitors,” “endothelin receptor antagonists” and “randomized controlled trial.” Both “AND” and “OR” as Boolean operators were used in order to be as exhaustive as possible. The database results were uploaded to reference management software (Endnote 20, Thomson Reuters, Philadelphia), where the inclusion and exclusion decisions were recorded. The specific retrieval strategy is shown in Appendix S1 in the supporting information, http://links.lww.com/MD/O55.

### 2.3. Eligibility criteria

According to PICOS principles, the studies involved must meet the conditions listed below: The study participants were diagnosed with COPD according to the Global Initiative for COPD (GOLD guidelines), which are forced expiratory volume in 1 second (FEV1)/ forced vital capacity (FVC) ratio < 70%. The assessment of airflow obstruction severity in COPD is based on the post-bronchodilator value of FEV1 (% reference): GLOD 1 (mild): FEV1 ≥ 80% predicted, GLOD 2 (moderate): 50% ≤ FEV1 < 80% predicted, GLOD 3 (severe): 30% ≤ FEV1 < 50% predicted and GLOD 3 (very severe): FEV1 < 30% predicted.^[14]^ The interventions were treatment with vasodilators, including ACEIs, ARBs, CCBs, nitrates, PDE-5 inhibitors, NO, prostacyclins and endothelin receptor antagonists. The patients in the control group received placebo or the usual care including oxygen therapy, ambroxol phlegm, β2 receptor agonist bronchodilator, inhaled corticosteroids anti-inflammatory, broad-spectrum antibiotics, and other comprehensive treatment. The studies were designed as randomized controlled trial studies that provided outcomes of blood gas analyses, lung function, and Georges Respiratory Questionnaire score (SGRQ) data. Exclusion criteria were the studies were conducted in cell assays or animal experiments; reviews, case reports, conference abstracts, letters, or editorials; duplicated publications or publications with incomplete data; and non-English manuscripts.

### 2.4. Study selection

A PRISMA flow diagram was used to summarize the study selection processes by 2 individual reviewers (HNX and QH).^[13]^ Duplicate articles were first eliminated from the initial database searches using the EndNote software package. Then, the additional duplicate entries were manually eliminated by comparing the authors, publication dates, and titles. Furthermore, the selected unduplicated studies were screened by 2 independent reviewers (HNX and QH) based on the title, abstract and quality of the full manuscript. In cases of conflict or disagreement, the eligibility of a study was discussed by all authors until a consensus was reached.

### 2.5. Data extraction

All data were extracted by 2 reviewers (HNX and QH) and were checked by the third reviewer (YYJ) to ensure accuracy. Data extracted included the authors, publication year, study design, disease, age, percentage of male participants, sample size, intervention, treatment duration, primary outcomes, secondary outcomes and any data from a subjective evaluation performed by the investigator or any other content that needed to be recorded.

### 2.6. Outcomes

A major function of lung is to provide optimal diffusion conditions for efficient oxygen exchange between the atmosphere and the blood stream.^[15]^ To assess the effect of vasodilators on pulmonary blood exchange function in COPD patients, carbon monoxide dispersion (DLCO) and 3 arterial blood gas indexes, including arterial carbon dioxide tension (PaCO_2_), arterial oxygen tension (PaO_2_), and arterial oxygen saturation (SaO_2_), were included as primary outcomes for this meta-analysis. The secondary outcomes include lung function and quality of life indicators. Pulmonary function testing is an important method to determine the level of airflow limitation for diagnosing and evaluating COPD patients. Meta-analyses were performed for 5 indicators, namely, FEV1, predicted percentage of forced expiratory volume in the first second (FEV1%), FEV1/FVC ratio, and *V*_*E*_*/V*_*CO2*_ slope. The total SGRQ score were used to evaluate quality of life in COPD patients.

### 2.7. Quality assessment

Two independent reviewers (YYJ and QH) assessed the risk of bias of the included studies using the Cochrane risk of bias tool^[16]^ based on the following 7 items: random sequence generation, allocation concealment, blinding of participants and personnel, blinding of outcome assessment, incomplete outcome data, selective reporting, and other sources of bias such as small sample size, baseline imbalance, conflicts of interest, etc.

### 2.8. Statistical analysis

Meta-analysis was performed using RevMan (version 5.4; The Nordic Cochrane Centre, The Cochrane Collaboration) and STATA 17 software. Continuous variables were analyzed using MD with the 95% CI upon the reported units, and a 2-sided *P* < .05 was considered statistically significant. We conducted a subgroup analysis according to the types of vasodilators and COPD patients with or without PH.

The interstudy heterogeneity was tested by Cochran *Q* (*P* < .10) and quantified by the *I*^2^ statistic, where an *I*^2^ ≥ 50% is considered to be evidence of considerable heterogeneity. A fixed-effect model and a random-effect model were used when *I*^2^ < 50% and *I*^2^ > 50% respectively. The random-effects model was preferentially chosen as the result if both random-effects model and fixed-effects model were implemented in the meta-analysis. Because of the clinical heterogeneity caused by different drugs, age, COPD staging, and other factors. Publication bias was assessed qualitatively by visual inspection of the funnel plot and quantitatively evaluated using the Egger and the Begg test. We performed sensitivity analyses by excluding trials that had a high risk of bias. *P *< .05 (two-tailed) was regarded as statistically significant.

## 3. Results

### 3.1. Studies selection

**Figure 1.**
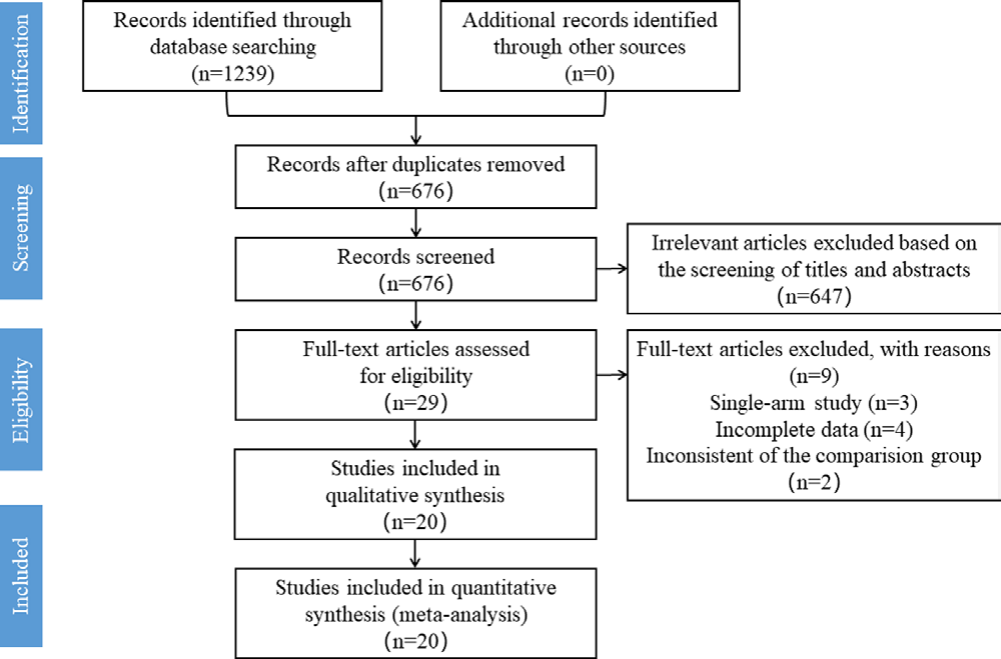
Preferred Reporting Items for Systematic Reviews and Meta-Analyses chart: the identification and selection of the studies for inclusion.

A total of 1239 studies were retrieved in the primary search. After removing 563 duplicated studies, 676 studies were remained for further screening. By screening titles and abstracts, 29 potentially relevant studies were retrieved for a full-text review. Moreover, 9 of them were excluded for the following reasons: 3 were single-arm studies, 4 had incomplete data, and 2 had inconsistent comparison groups. Finally, a total of 20 studies met the eligibility criteria and were included in this meta-analysis. The Preferred Reporting Items for Systematic Reviews and Meta-Analyses flowchart of the study selection is shown in Figure 1.

### 3.2. Study characteristics

The study sample included 986 participants from 20 randomized controlled trials (RCTs),^[17–36]^ including 6 crossover RCTs.^[21,24,26,30–32]^ The main characteristics of the included studies are shown in Table 1, and the outcome data from each included study are shown in Table 2.

**Table 1:** Basic data of the included studies.

Study	Year	Study design	Disease	GOLD Stage	Age	Male%	Sample size	Intervention	Outcome
Maron et al^[17]^	2022	RCT	COPD‐PH	II to IV	68 ± 7.6	NA	24	Tadalafil (40 mg/d) vs placebo	i
Andreas et al^[18]^	2006	RCT	COPD	III to IV	30 to 80	71.10%	60	Irbesartan (150 mg/d) vs placebo	bcehi
Tuyet et al^[19]^	2022	RCT	COPD-PH	–	56 to 75	76.70%	30	Inhaled NO vs conventional therapy	bcd
Robert et al^[20]^	2008	RCT	COPD	–	≥40	50.00%	106	Losartan (100 mg/d) vs placebo	e
Di Marco et al^[21]^	2010	RCT crossover	COPD	II to IV	69 ± 11	89.00%	18	Enalapril (10 mg/d) vs placebo	bceh
Shrikrishna et al^[22]^	2014	RCT	COPD	-	65 ± 8	52.50%	80	Fosinopril (10 mg/d) vs placebo	abcfi
Curtis et al^[23]^	2016	RCT	COPD	II to IV	67 ± 8	52.30%	65	Enalapril (10 mg/d) vs placebo	abcefi
Dujić et al^[24]^	1991	RCT crossover	COPD-PH	-	41 to 68	NA	17	Nifedipine (10 or 20 mg/d) vs placebo	abcf
Cheng et al^[25]^	1997	RCT	COPD-Chronic pulmonary heart disease	-	20 to 85	61.00%	10	Nifedipine controlled release tablet (20 mg/d) vs placebo	bcdef
Lederer et al^[26]^	2012	RCT crossover	COPD – emphysema	II to IV	66 ± 4	80.00%	10	Sildenafil (75 mg/d) vs placebo	bcegi
Andrew et al	2014	RCT	COPD	II to IV	35 to 85	68.30%	120	Tadalafil (10 mg/d) vs placebo	aef
Bozorgmehr et al^[28]^	2020	RCT	COPD	II to IV	64.95 ± 8.99	82.50%	40	Verapamil (10 mg/d) vs placebo	efg
Lee et al^[29]^	2004	RCT	COPD	II to IV	54 to 74	95.20%	21	Beraprost Sodium (60–180 μg/d) vs placebo	abcdefg
Oliver et al^[30]^	1989	RCT crossover	COPD	IV	53 to 72	55.60%	9	Captopril (50 mg/d) vs placebo	bcf
Patrizio et al	2017	RCT	COPD-PH	IV	40 to 80	75.00%	28	Sildenafil (20 mg/d) vs placebo	abcfg
Phillips et al^[31]^	2021	RCT crossover	COPD	I	48 to 75	63.30%	30	Inhaled nitric oxide (40ppm) vs placebo	h
Boeck et al^[32]^	2012	RCT crossover	COPD	–	73.2 ± 6.7	62.50%	16	Iloprost (10 or 20 mg/d) vs placebo	bch
Morrell et al^[35]^	2005	RCT	COPD-PH	–	68 ± 8.4	47.50%	40	Losartan (50 mg/d) vs placebo	i
Vonbank et al^[36]^	2003	RCT	COPD	–	62 ± 7.6	67.50%	40	Inhaled NO (900 ppm) vs oxygen	bceg
Stolz et al^[34]^	2008	RCT	COPD	IV	65 ± 7.9 69.5 ± 8.8	60%	30	Bosentan (125–250 mg/d) vs placebo	aefg

DLCO = carbon monoxide dispersion, FEV1 = forced expiratory volume in the first second, FEV1% = predicted percentage of forced expiratory volume in the first second, FEV1/FVC = forced expiratory volume in the first second/forced vital capacity ratio, PaCO_2_ = arterial carbon dioxide tension, PaO_2_ = arterial oxygen tension, PH = pulmonary hypertension, RCT = randomized controlled trial, SaO_2_ = arterial oxygen saturation, SGRQ = Georges respiratory questionnaire score, V_*E*_*/V*_*CO2*_ slope = ventilatory equivalent to CO_2_ production slope.

**Table 2:** Efficacy outcomes of included RCTs.

Study	Primary outcomes (vasodilators/control)					Secondary outcomes (vasodilators/control)				
	No. of Patients	DLCO (% predicted)	PaCO_2_ (kpa)	PaO_2_ (kpa)	SaO_2_(%)	FEV1 (L/s)	FEV1 (% predicted)	FEV1/FVC (%)	V_*E*_*/V*_*CO2*_ slope	SGRQ
Maron 2022^[17]^	9/15	NA	NA	NA	NA	NA	NA	NA	NA	45.3 ± 10.2/
										57.7 ± 11.3
Andreas 2006^[18]^	26/24	NA	5.7 ± 0.5/	8.9 ± 1.6/	NA	0.87 ± 0.3/	NA	NA	24.1 ± 14.3/	47.8 ± 11.65/
			5.9 ± 0.5	8.3 ± 1.6		0.95 ± 0.6			23.4 ± 16.1	52.5 ± 18.25
Tuyet 2022^[19]^	15/15	NA	5.8 ± 0.1/	10.4 ± 1.9/	94.9 ± 1.4/	NA	NA	NA	NA	NA
			5.9 ± 0.1	9.7 ± 0.4	92.2 ± 2.0					
Robert 2008^[20]^	23/20	NA	NA	NA	NA	1.48 ± 0.68/	NA	NA	NA	NA
						1.36 ± 0.59				
Di Marco 2010^[21]^	18	NA	5.5 ± 0.5/	9.9 ± 0.7/	NA	1.30 ± 0.36/	NA	NA	32.8 ± 9.4/	NA
			5.6 ± 0.5	9.9 ± 0.7		1.32 ± 0.34			34.4 ± 8.5	
Shrikrishna 2014^[22]^	31/36	44.2 ± 22.51/	5.1 ± 0.6/	9.6 ± 1.7/	NA	NA	46.1 ± 21.71/	NA	NA	48.8 ± 17.26/
		44.5 ± 23.4	5.3 ± 0.6	9.5 ± 1.8			42.7 ± 22.2			50.80 ± 21.60
Curtis 2016^[23]^	31/34	49.14 ± 30.75/	4.88 ± 0.62/	10.40 ± 1.62/	NA	1.09 ± 0.72/	48.1 ± 29.55/	NA	NA	42.12 ± 21.43/
		52.75 ± 30.41	4.62 ± 0.65	10.42 ± 1.56		1.29 ± 0.71	51.62 ± 27.18			43.11 ± 24.19
Dujić 1991^[24]^	17/15	101.2 ± 14.2/	5.45 ± 0.55/	12.95 ± 8.41/	NA	NA	53.1 ± 14.68/	NA	NA	NA
		100.5 ± 12.5	5.6 ± 0.6	12.7 ± 1.0			52.3 ± 15.70			
Cheng 1997^[25]^	102/100	NA	5.56 ± 0.71/	9.41 ± 0.93/	93.80 ± 1.94/	0.92 ± 0.42/	46.22 ± 8.68/	NA	NA	NA
			5.51 ± 0.73	9.65 ± 1.19	93.18 ± 1.58	0.96 ± 0.53	46.84 ± 9.86			
Lederer 2012^[26]^	10	NA	5.60 ± 0.65/	9.73 ± 1.21/	NA	1.10 ± 0.47/	NA	39 ± 11.54/	NA	39 ± 20.27/
			6.53 ± 0.65	10.27 ± 1.21		1.10 ± 0.38		40 ± 9.35		36 ± 20.27
Andrew 2014	56/57	41.2 ± 16.40/	NA	NA	NA	1.05 ± 0.38/	40.10 ± 15.40/	NA	NA	NA
		34.20 ± 11.20				0.99 ± 0.40	38.90 ± 15.20			
Bozorgmehr 2020^[28]^	20/20	NA	NA	NA	NA	1.90 ± 0.40/	45.40 ± 13.40/	59.30 ± 11.10/	NA	NA
						1.09 ± 0.60	41.50 ± 19.10	58.50 ± 12.30		
Lee 2004^[29]^	11/10	75.50 ± 15.40/	5.48 ± 0.77/	10.07 ± 2.24/	93.9 ± 5.6/	1.00 ± 0.34/	40.00 ± 11.60/	33.80 ± 10.10/	NA	NA
		86.50 ± 18.30	5.47 ± 0.52	11.32 ± 1.85	96.2 ± 1.6	0.95 ± 0.34	36.00 ± 10.50	35.40 ± 6.70		
Oliver 1989^[30]^	9	NA	5.67 ± 1.14/	8.34 ± 2.34/	NA	NA	32.90 ± 8.40/	NA	NA	NA
			5.90 ± 1.02	8.08 ± 1.95			32.10 ± 9.30			
Patrizio 2017	10/18	35.02 ± 17.60/	5.63 ± 0.88/	9.21 ± 1.84/	NA	NA	54.60 ± 22.70/	51 ± 13/	NA	NA
		31.27 ± 16.70	5.50 ± 0.85	9.36 ± 1.68			45.63 ± 22.48	57 ± 13		
Phillips 2021^[31]^	15/15	NA	NA	NA	NA	NA	NA	NA	36.6 ± 3.5/	NA
									36.4 ± 4.3	
Boeck 2012^[32]^	16	NA	6.01 ± 1.23/	7.33 ± 1.55/	NA	NA	NA	NA	37.8 ± 7.5/	NA
			5.86 ± 1.39	7.84 ± 2.20					38.5 ± 8.5	
Morrell 2005^[35]^	20/20	NA	NA	NA	NA	NA	NA	NA	NA	60 ± 15/
										66 ± 19
Vonbank 2003^[36]^	20/20	NA	6.7 ± 1.1/	10.1 ± 1.6/	NA	1.07 ± 0.4/	NA	45.5 ± 13.3/	NA	NA
			6.7 ± 1.1	10.6 ± 1.9		1.28 ± 0.7		46.1 ± 13.2		
Stolz 2008^[34]^	10/20	35 ± 18/	NA	NA	NA	0.86 ± 0.26/	35 ± 12/	31 ± 9/	NA	NA
		42 ± 13				1.07 ± 0.38	42 ± 14	35 ± 11		

### 3.3. Risk of bias

**Figure 2.**
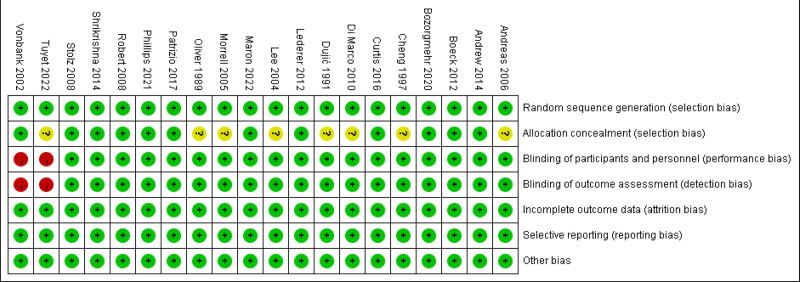
Risk of bias summary.

**Figure 3.**
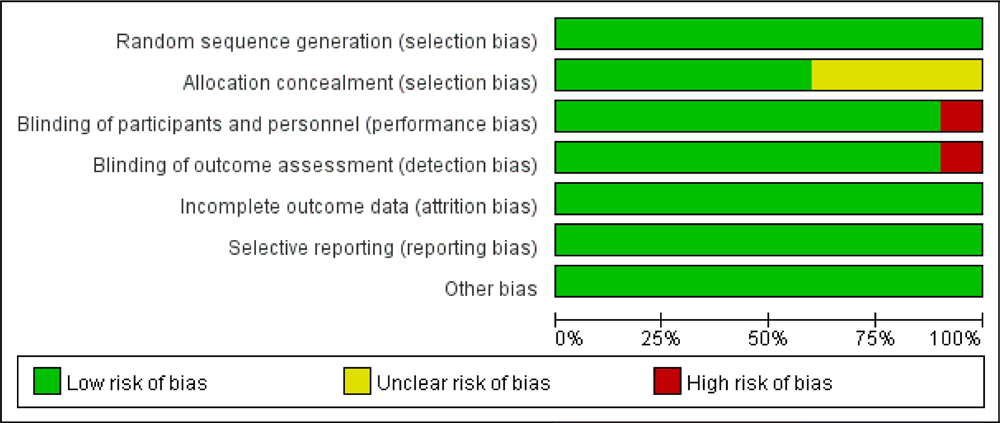
Risk of bias graph.

We evaluated the risk of bias in each of the 20 RCTs, and the details are shown in Figures 2 and 3. Out of the 20 studies, 11 were deemed to be of high-quality.^[17,20,22,23,26–28,31–34]^ All 20 studies were considered to have low risk for random sequence generation (selection bias), incomplete outcome data (attrition bias), selective reporting (reporting bias), blinding of outcome assessment (detection bias) and other biases. Eight studies^[18,19,21,24,25,29,30,35]^ could not be assessed for allocation concealment (selection bias) due to incomplete information. Two studies^[19,36]^ were considered to have high risk due to their methods regarding the blinding of participants and personnel (performance bias).

### 3.4. Synthesis of the results

#### 3.4.1. Mean change in carbon monoxide dispersion

**Figure 4.**
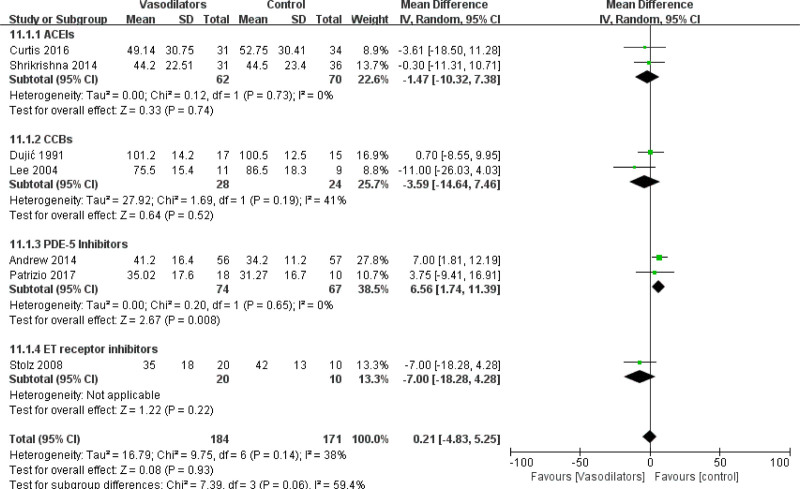
Forest plot for subgroup analysis of DLCO by vasodilators.

**Figure 5.**
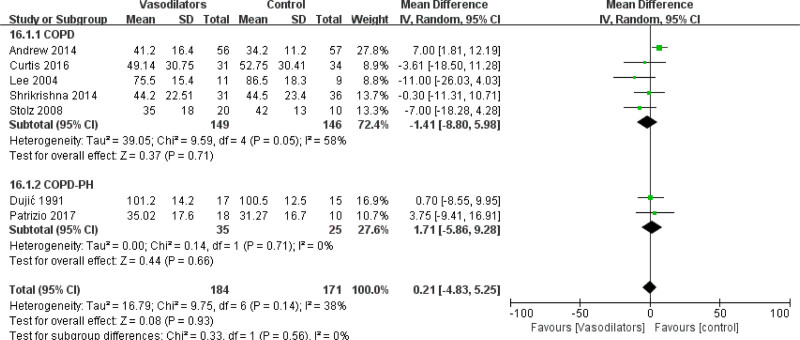
Forest plot for subgroup analysis of DLCO by COPD with or without PH.

The pooled MD in DLCO after the intervention was 0.21 [95% CI (−4.83, 5.25)], as shown in Figure 4. The results indicated that vasodilators have no effect on DLCO (*P* = .93). However, in subgroup analysis, we found that treatment with PDE-5 inhibitors but not with other drugs could improve DLCO in patients with COPD (MD = 6.56 [95% CI (1.74, 11.39)], *P* = .008), as shown in Figure 4. In addition, we divided the pooled data into 2 subgroups by COPD patients with or without PH and no significant differences were found upon vasodilator treatment in these subgroups, as shown in Figure 5.

#### 3.4.2. Mean change in arterial carbon dioxide tension and arterial oxygen tension

**Figure 6.**
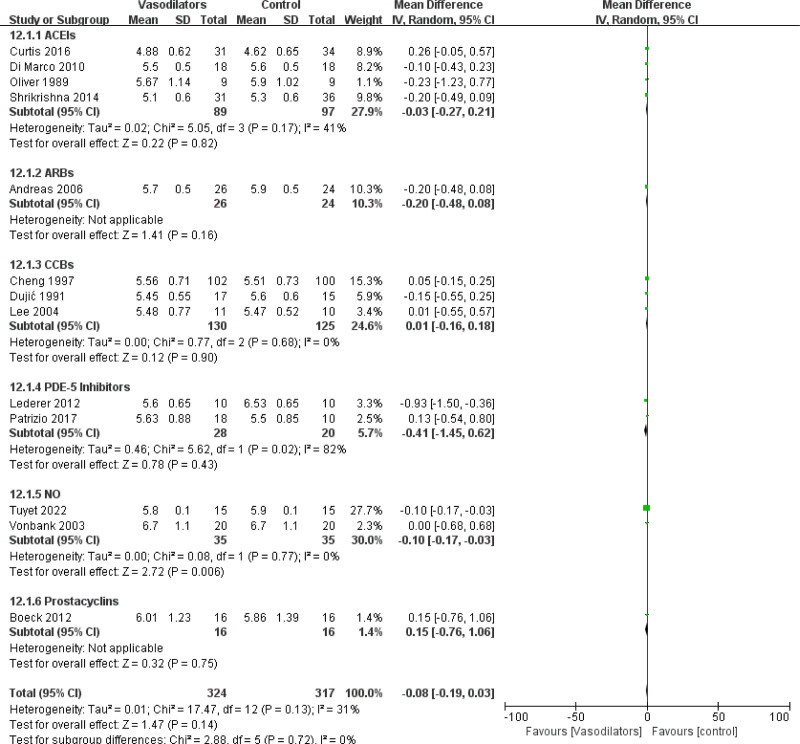
Forest plot for subgroup analysis of PaCO_2_ by vasodilators.

**Figure 7.**
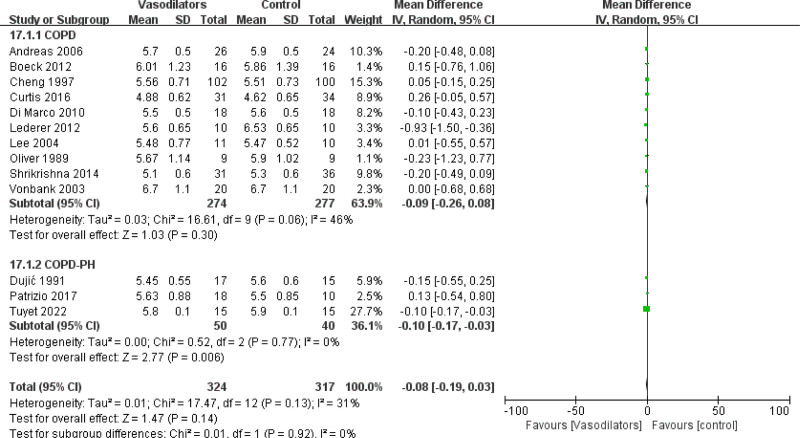
Forest plot for subgroup analysis of PaCO_2_ by COPD with or without PH.

PaCO_2_ and PaO_2_ were measured in 13 studies (n = 596 patients), and the results indicated that treatment with NO could obviously reduce PaCO_2_ in patients with COPD (MD = −0.10 [95% CI (−0.17, −0.03)], *P* = .006). Other drugs had no significant effects on PaCO_2_, as shown in Figure 6. In COPD with or without PH subgroup analysis, vasodilators could reduce PaCO_2_ in COPD with PH patients (MD = −0.10 [95% CI (−0.17, −0.03)], *P* = .006), as shown in Figure 7. However, CCBs seemed to have an aggravating effect on PaO_2_ (MD = −0.23 [95% CI (−0.46, 0.01)], *P* = .06) in subgroup of vasodilators, as shown in Figure S1, Supplemental Digital Content, http://links.lww.com/MD/N885. The impact of vasodilators on PaO_2_ was inconspicuous in subgroup of COPD with PH (MD = −0.12 [95% CI (−0.30, 0.06)], *P* = .19), as shown in Figure S2, Supplemental Digital Content, http://links.lww.com/MD/N885.

#### 3.4.3. Mean change in arterial oxygen saturation

The pooled results of 3 RCTs from 253 patients were used to evaluate SaO_2_. The results showed that treatment with NO but not with CCBs could increase SaO_2_ in COPD patients with PH (MD = 2.70 [95% CI (1.46, 3.94)], *P* < .0001), as shown in Figures S3 and S4, Supplemental Digital Content, http://links.lww.com/MD/N885.

#### 3.4.4. Mean change in forced expiratory volume in the first second

The pooled results of 11 RCTs from 712 patients were used to evaluate FEV1. The results indicated that compared with placebo or other treatments vasodilator treatment did not improve FEV1 (MD = 0.02 [95% CI (−0.11, 0.16)], *P *= .74), as shown in Figure S5, Supplemental Digital Content, http://links.lww.com/MD/N885. An exploratory sensitivity analysis revealed that the study by Bozorgmehr et al^[28]^ was a likely source of this heterogeneity, as its exclusion from the meta-analysis resulted in a revised *I*^2^ of 0%, but it did not affect the overall result, shown in Figure S6, Supplemental Digital Content, http://links.lww.com/MD/N885. There might be 2 factors contributed to this observation: One was that the study had a short dosing period with only 10 minutes, which represent only a short-term effect, and the other was that the vasodilator was administered by inhalation, which might directly affect the lung area more efficiently.

#### 3.4.5. Mean change in predicted percentage of forced expiratory volume in the first second

The pooled difference in the mean change in FEV1% predicted in the 10 RCTs from 552 patients showed that vasodilators had no effect on FEV1% predicted compared with placebo (MD = 0.07 [95% CI (−1.90, 2.05)], *P* = .94), as shown in Figure S7, Supplemental Digital Content, http://links.lww.com/MD/N885.

#### 3.4.6. Mean change in forced expiratory volume in the first second/forced vital capacity

The pooled difference in the mean change in FEV1/FVC in the 5 RCTs suggested that there were no FEV1/FVC differences between vasodilators and placebo (MD = 0.70 [95% CI (−4.02, 5.42)], *P* = .77), as shown in Figure S8, Supplemental Digital Content, http://links.lww.com/MD/N885.

#### 3.4.7. *Mean change in V*_*E*_*/V*_*CO2*_

Three studies provided numerical data for *V*_*E*_*/V*_*CO2*_ were included in the meta-analysis. The pooled result showed that vasodilator treatment did not improve *V*_*E*_*/V*_*CO2*_, (MD = −0.17 [95% CI (−2.39, 2.05)], *P* = .88), as shown in Supplementary Figure S9, Supplemental Digital Content, http://links.lww.com/MD/N885.

#### 3.4.8. *Mean change in* Georges respiratory questionnaire score

**Figure 8.**
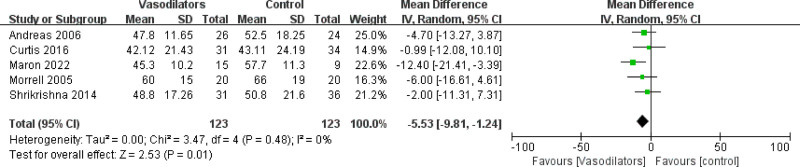
Forest plot of SGRQ.

The meta-analysis of 5 studies showed that treatment with vasodilators significantly reduced the total SGRQ score (MD = −5.53 [95% CI (−9.81, −1.24)], *P *= .01), as shown in Figure 8. The results indicated that treatment with vasodilators could reduce the total SGRQ score in patients with COPD.

#### 3.4.9. Publication bias

The results of bias assessment are summarized in Figure S10A–I, Supplemental Digital Content, http://links.lww.com/MD/N885. Some metrics SO_2_, FEV1, FEV1/FVC, and *V*_*E*_*/V*_*CO2*_ funnel plots are asymmetric and suggesting the presence of publication bias, which may be attributed in part to the small number of studies and patients, the results of the Egger or Begg quantitative statistics showed that there was no significant publication bias in any of the studies (all *P* > .05).

## 4. Discussion

It has been proven that microvascular dysfunction, including microvascular loss and reduced PMBF,^[37]^ is an early event during the progression of COPD;^[38]^ however, whether microvascular dysfunction is reversible in patients with COPD is still unclear.^[39]^ Given that vasodilators may contribute to improving vascular dysfunction, as far as we know, no systematic reviews focused on the effect of vasodilators on COPD have yet been published. In the present study, we investigated the effects of 8 groups of vasodilators on COPD from multiple perspectives. This meta-analysis summarized and compared 20 studies involving 986 patients. Our research analyzed the DLCO, PaCO_2_ and PaO_2_, SaO_2_, FEV1, FEV1% predicted, FEV1/FVC, *V*_*E*_*/V*_*CO2*_, and SGRQ scores.

### 4.1. Treatment with vasodilators has certain benefits in improving gas-blood exchange function among chronic obstructive pulmonary disease patients

The pulmonary gas-blood barrier is a membrane that is used to exchange oxygen in the alveoli and carbon dioxide in the capillaries of the lungs.^[40]^ DLCO is a gas exchange index that reflects the complex interplay of the alveolar capillary barrier and describes the extent to which inhaled gases diffuse across the alveolar, capillary, erythrocyte membranes and ultimately react with hemoglobin.^[41]^ The decrease in DLCO among patients with COPD is usually associated with emphysema. Emphysema affects the distal bronchioles and causes occlusions of the peripheral alveolar vessels, thereby resulting in reduced capillary blood flow and a reduction in the alveolar capillary surface area available for gas exchange.^[42]^ Although our meta-analysis results initial revealed that the 8 types of vasodilator drugs had no significant effect on DLCO, which may be due to the different mechanisms of action of the drugs, subgroup analysis showed that PDE-5 inhibitors could effectively improve DLCO indicating the therapeutic potential of PDE-5 inhibitors for COPD. Consistent to this hypothesis, Snyder et al^[43]^ found that sildenafil is preserving the integrity of the alveolar–capillary membrane during hypoxic exposure. Mechanistically, a PDE-5 inhibitor works by blocking the degradative action of PDE-5 on cyclic guanosine monophosphate (cGMP) and subsequently enhancing nonmedicated vasodilation.^[44]^ Furthermore, we found that vasodilators did not benefit DLCO in COPD-PH patients.

Blood gas analysis such as PaCO_2_ is an important parameter to evaluate lung gas exchange function. Our results suggested that inhaled NO plays an important role in reducing PaCO_2_ in COPD patients. Inhaled NO is a selective pulmonary vasodilator that induces relaxation of smooth muscle cells in the vasculature. NO has the unique ability to induce pulmonary vasodilatation specifically in the portions of the lung with adequate ventilation, thereby improving oxygenation of blood and decreasing intrapulmonary right to left shunting.^[45]^

PH, characterized by pulmonary vascular injury/remodeling, is a common complication of COPD.^[46]^ Multiple vasodilators have been proven to be effective in improving haemodynamic in patients with PH.^[47]^ Our meta-analysis results indicated that vasodilators reduce PaCO_2_ but did not significantly affect the PaO_2_ and SaO_2_ in COPD-PH patients, suggesting that vasodilators are beneficial for the recovery of blood exchange function in COPD-PH patients. To prove this hypothesis, additional large number of researches are needed.

### 4.2. Treatment with vasodilators has no effect on improving airflow restriction among chronic obstructive pulmonary disease patients

Our meta-analysis indicated that after vasodilator treatment there were no significant improvements in FEV1, FEV1% predicted and FEV1/FVC in COPD patients. Although improving lung function is not an objective of COPD management, it is normally the primary end-point most frequently used by regulatory authorities in interpreting drug efficacy in COPD trials.^[48]^ A possible explanation here might be that microvascular dysfunction is an early event in the natural history of COPD,^[38]^ and it is an open issue whether microvascular dysfunction is still reversible in patients with advanced COPD.^[39]^

*V*_*E*_*/V*_*CO2*_ is clinically important because it independently predicts mortality in COPD.^[49]^ Previous research has demonstrated an elevated *V*_*E*_*/V*_*CO2*_ during exercise, secondary to increased dead space ventilation in mild COPD.^[50]^ Pulmonary microvascular dysfunction and the corresponding capillary hypoperfusion are potential mechanisms for the increased dead space. In this review, there was no significant improvement in *V*_*E*_*/V*_*CO2*_ between the 2 groups, indicating that not all of the vasodilators acting directly on the pulmonary microvasculature.

### 4.3. Treatment with vasodilators has certain benefits in improving quality of life among chronic obstructive pulmonary disease patients

SGRQ scores are a well-established disease-specific instrument to measure quality of life for asthma and COPD. The results of our meta-analysis showed that vasodilators can improve the total SGRQ scores. This means that vasodilators have a certain effect on improving the quality of life in patients with COPD, which is probably due to severe pulmonary vascular remodeling with prominent narrowing of the pulmonary vascular lumen, resembling the characteristics of pulmonary vascular remodeling in PH in patients with COPD.

### 4.4. Prospects for chronic obstructive pulmonary disease treatment

In COPD patients, changes in the large arteries are typical manifestations of atherosclerosis; severe lesions, which range from stenosis to complete occlusion and from vulnerable plaques to plaque rupture and distal embolization, are normally studied in the coronary arteries, internal artery, carotid artery an d other large vessels.^[7]^ However, lesions in the microcirculation which may preceded the lesions in the large arteries are often overlooked. It has been demonstrated that the complications of atherosclerosis within large arteries and subsequent organ failure is associated with the degree of microvascular damage.^[29]^ In addition, growing evidence have shown that endothelial dysfunction within the pulmonary microvasculature is an early feature of COPD.^[51]^ Seimetz et al^[52]^ found that changes in the structure and function of mouse pulmonary vessels after tobacco smoke exposure occurred prior to emphysema. A MESA COPD study suggested that the number of microparticles that reflect apoptosis of CD31^+^ endothelial cells was significantly increased and negatively correlated with PMBF in mild patients with COPD,^[53]^ suggesting that microvascular endothelial injury presented in early-stage of COPD might lead to abnormal PMBF. Therefore, new drugs targeting the pulmonary microvasculature during the early stage of the disease may efficiently prevent the progression of COPD.

The therapeutic effects of long-term application of vasodilators in the treatment of COPD are unclear. The use of ACEIs, ARBs, CCBs, NO, prostacyclins, and endothelin receptor antagonists did not result in any improvement in lung function parameters. However, positive effects of PDE-5 inhibitors and NO in improving gas-blood exchange function and vasodilators can improve quality of life in patients with COPD encourage us to make the prediction that vasodilators can be used in concert. In addition, evidence is inadequate to support the potential of vasodilators in improving exercise capacity and breathlessness in COPD patients. Since there are currently limitations to studies concerning the therapeutic effects of vasodilators on COPD, further high-quality RCTs are needed to evaluate the efficacy of vasodilators for COPD patients.

## 5. Limitations

There are some limitations of this study. First, only 986 COPD subjects were included. The small sample size might weaken the statistical power of the pooled results. Second, the pooled estimates in this meta-analysis were not based on adjustment by potential confounding factors, such as age, sex, smoking history, nationality, combined therapies, dose and types of vasodilators, which might influence the stringency of the pooled results.

## Author contributions

**Conceptualization:** Zhenhua Jia.

**Data curation:** Hui Qi, Yujie Yin.

**Formal analysis:** Ningxin Han.

**Investigation:** Hui Qi, Yujie Yin, Yunlong Hou.

**Methodology:** Ningxin Han.

**Supervision:** Yunlong Hou, Zhenhua Jia.

**Validation:** Yi Liu, Peipei Jin.

**Writing – original draft:** Ningxin Han.

**Writing – review & editing:** Hui Qi.

## Supplementary Material
