# Supplementary material for: The effectiveness of vasodilators on chronic obstructive pulmonary disease: A systematic review and meta-analysis
Source: Medicine (Baltimore). 2024 Nov 15;103(46):e39794. doi: 10.1097/MD.0000000000039794 (PMC11576023; doi:10.1097/MD.0000000000039794)
Supplement: Supplementary file 1 [file medi-103-e39794-s001.docx]

**Appendix S1: Search strategies**

PubMed Search

Query

((("Pulmonary Disease, Chronic Obstructive"[Mesh]) OR ((((((((((Chronic Obstructive Lung Disease[Title/Abstract]) OR (Chronic Obstructive Pulmonary Diseases[Title/Abstract])) OR (COAD[Title/Abstract])) OR (COPD[Title/Abstract])) OR (Chronic Obstructive Airway Disease[Title/Abstract])) OR (Chronic Obstructive Pulmonary Disease[Title/Abstract])) OR (Airflow Obstruction, Chronic[Title/Abstract])) OR (Airflow Obstructions, Chronic[Title/Abstract])) OR (Chronic Airflow Obstructions[Title/Abstract])) OR (Chronic Airflow Obstruction[Title/Abstract]))) AND (((((((((("Angiotensin-Converting Enzyme Inhibitors"[Mesh]) OR ((((((((((((((((((((((((((((Angiotensin Converting Enzyme Inhibitors[Title/Abstract]) OR (Enzyme Inhibitors, Angiotensin-Converting[Title/Abstract])) OR (Inhibitors, Angiotensin-Converting Enzyme[Title/Abstract])) OR (Inhibitors, Angiotensin Converting Enzyme[Title/Abstract])) OR (Inhibitors, Kininase II[Title/Abstract])) OR (Kininase II Antagonists[Title/Abstract])) OR (Kininase II Inhibitors[Title/Abstract])) OR (Angiotensin-Converting Enzyme Antagonists[Title/Abstract])) OR (Angiotensin Converting Enzyme Antagonists[Title/Abstract])) OR (Enzyme Antagonists, Angiotensin-Converting[Title/Abstract])) OR (Kininase II Inhibitor[Title/Abstract])) OR (II Inhibitor, Kininase[Title/Abstract])) OR (Inhibitor, Kininase II[Title/Abstract])) OR (Antagonists, Kininase II[Title/Abstract])) OR (Inhibitors, ACE[Title/Abstract])) OR (ACE Inhibitors[Title/Abstract])) OR (Angiotensin I-Converting Enzyme Inhibitors[Title/Abstract])) OR (Angiotensin I Converting Enzyme Inhibitors[Title/Abstract])) OR (Angiotensin Converting Enzyme Inhibitor[Title/Abstract])) OR (ACE Inhibitor[Title/Abstract])) OR (Inhibitor, ACE[Title/Abstract])) OR (Angiotensin I-Converting Enzyme Inhibitor[Title/Abstract])) OR (Angiotensin I Converting Enzyme Inhibitor[Title/Abstract])) OR (Angiotensin-Converting Enzyme Inhibitor[Title/Abstract])) OR (Enzyme Inhibitor, Angiotensin-Converting[Title/Abstract])) OR (Inhibitor, Angiotensin-Converting Enzyme[Title/Abstract])) OR (Antagonists, Angiotensin-Converting Enzyme[Title/Abstract])) OR (Antagonists, Angiotensin Converting Enzyme[Title/Abstract]))) OR (("Angiotensin Receptor Antagonists"[Mesh]) OR ((((((((((((((Antagonists, Angiotensin Receptor[Title/Abstract]) OR (Receptor Antagonists, Angiotensin[Title/Abstract])) OR (Angiotensin Receptor Blockers[Title/Abstract])) OR (Receptor Blockers, Angiotensin[Title/Abstract])) OR (Angiotensin Receptor Blocker[Title/Abstract])) OR (Blocker, Angiotensin Receptor[Title/Abstract])) OR (Receptor Blocker, Angiotensin[Title/Abstract])) OR (Angiotensin Receptor Antagonist[Title/Abstract])) OR (Antagonist, Angiotensin Receptor[Title/Abstract])) OR (Receptor Antagonist, Angiotensin[Title/Abstract])) OR (Angiotensin II Receptor Antagonists[Title/Abstract])) OR (Angiotensin II Receptor Blockers[Title/Abstract])) OR (Angiotensin II Receptor Antagonist[Title/Abstract])) OR (Angiotensin II Receptor Blocker[Title/Abstract])))) OR (("Calcium Channel Blockers"[Mesh]) OR (((((((((((((((((((((((((Blockers, Calcium Channel[Title/Abstract]) OR (Calcium Channel Blocking Drug[Title/Abstract])) OR (Channel Blockers, Calcium[Title/Abstract])) OR (Calcium Channel Antagonists[Title/Abstract])) OR (Antagonists, Calcium Channel[Title/Abstract])) OR (Calcium Channel Blocking Drugs[Title/Abstract])) OR (Calcium Channel Antagonist[Title/Abstract])) OR (Antagonist, Calcium Channel[Title/Abstract])) OR (Channel Antagonist, Calcium[Title/Abstract])) OR (Calcium Channel Blocker[Title/Abstract])) OR (Blocker, Calcium Channel[Title/Abstract])) OR (Channel Blocker, Calcium[Title/Abstract])) OR (Calcium Antagonists, Exogenous[Title/Abstract])) OR (Antagonists, Exogenous Calcium[Title/Abstract])) OR (Exogenous Calcium Inhibitor[Title/Abstract])) OR (Calcium Inhibitor, Exogenous[Title/Abstract])) OR (Inhibitor, Exogenous Calcium[Title/Abstract])) OR (Exogenous Calcium Inhibitors[Title/Abstract])) OR (Calcium Inhibitors, Exogenous[Title/Abstract])) OR (Exogenous Calcium Antagonists[Title/Abstract])) OR (Exogenous Calcium Blockaders[Title/Abstract])) OR (Exogenous Calcium Blockader[Title/Abstract])) OR (Blockader, Exogenous Calcium[Title/Abstract])) OR (Calcium Blockader, Exogenous[Title/Abstract])) OR (Calcium Blockaders, Exogenous[Title/Abstract])))) OR (("Nitroglycerin"[Mesh]) OR ((((Glyceryl Trinitrate[Title/Abstract]) OR (Trinitrate, glycerol[Title/Abstract])) OR (Nitrospan[Title/Abstract])) OR (Nitrostat[Title/Abstract])))) OR (("Phosphodiesterase 3 Inhibitors"[Mesh]) OR (((((((((((((Inhibitors, Phosphodiesterase 3[Title/Abstract]) OR (Phosphodiesterase Type 3 Inhibitor[Title/Abstract])) OR (PDE3 Inhibitor[Title/Abstract])) OR (Inhibitor, PDE3[Title/Abstract])) OR (Phosphodiesterase Type 3 Inhibitors[Title/Abstract])) OR (PDE-3 Inhibitors[Title/Abstract])) OR (Inhibitors, PDE-3[Title/Abstract])) OR (PDE 3 Inhibitors[Title/Abstract])) OR (PDE3 Inhibitors[Title/Abstract])) OR (Inhibitors, PDE3[Title/Abstract])) OR (Phosphodiesterase 3 Inhibitor[Title/Abstract])) OR (3 Inhibitor, Phosphodiesterase[Title/Abstract])) OR (Inhibitor, Phosphodiesterase 3[Title/Abstract])))) OR (("Nitric Oxide"[Mesh]) OR (((((((((((Oxide, Nitric[Title/Abstract]) OR (Nitrogen Monoxide[Title/Abstract])) OR (Monoxide, Nitrogen[Title/Abstract])) OR (Nitric Oxide, Endothelium-Derived[Title/Abstract])) OR (Endothelium-Derived Nitric Oxide[Title/Abstract])) OR (Nitric Oxide, Endothelium Derived[Title/Abstract])) OR (Endogenous Nitrate Vasodilator[Title/Abstract])) OR (Nitrate Vasodilator, Endogenous[Title/Abstract])) OR (Vasodilator, Endogenous Nitrate[Title/Abstract])) OR (Mononitrogen Monoxide[Title/Abstract])) OR (Monoxide, Mononitrogen[Title/Abstract])))) OR (("Epoprostenol"[Mesh]) OR ((((((((Epoprostanol[Title/Abstract]) OR (Prostaglandin I2[Title/Abstract])) OR (Prostacyclin[Title/Abstract])) OR (Prostaglandin I(2[Title/Abstract]))) OR (Veletri[Title/Abstract])) OR (Epoprostenol Sodium[Title/Abstract])) OR (Epoprostenol Sodium Salt, (5Z,9alpha,11alpha,13E,15S)-Isomer[Title/Abstract])) OR (Flolan[Title/Abstract])))) OR (endothelin-1 receptor antagonist)) OR (("Phosphodiesterase 5 Inhibitors"[Mesh]) OR ((((((((((((((((Inhibitors, Phosphodiesterase 5[Title/Abstract]) OR (Phosphodiesterase 5 Inhibitor[Title/Abstract])) OR (5 Inhibitor, Phosphodiesterase[Title/Abstract])) OR (Inhibitor, Phosphodiesterase 5[Title/Abstract])) OR (Phosphodiesterase Type 5 Inhibitor[Title/Abstract])) OR (PDE5 Inhibitors[Title/Abstract])) OR (Inhibitors, PDE5[Title/Abstract])) OR (Phosphodiesterase Type 5 Inhibitors[Title/Abstract])) OR (PDE-5 Inhibitor[Title/Abstract])) OR (Inhibitor, PDE-5[Title/Abstract])) OR (PDE 5 Inhibitor[Title/Abstract])) OR (PDE5 Inhibitor[Title/Abstract])) OR (Inhibitor, PDE5[Title/Abstract])) OR (PDE-5 Inhibitors[Title/Abstract])) OR (Inhibitors, PDE-5[Title/Abstract])) OR (PDE 5 Inhibitors[Title/Abstract]))))) OR ((((((((((((((((("Endothelin Receptor Antagonists"[MeSH Terms] OR ("antagonists endothelin receptor"[Title/Abstract] OR "endothelin antagonists"[Title/Abstract] OR "antagonists endothelin"[Title/Abstract] OR "endothelin receptor antagonist"[Title/Abstract] OR (("Antagonist"[All Fields] OR "antagonists and inhibitors"[MeSH Subheading] OR ("Antagonists"[All Fields] AND "inhibitors"[All Fields]) OR "antagonists and inhibitors"[All Fields] OR "Antagonists"[All Fields]) AND "endothelin receptor"[Title/Abstract]) OR "receptor antagonist endothelin"[Title/Abstract] OR "endothelin antagonist"[Title/Abstract] OR "antagonist endothelin"[Title/Abstract])))))) AND ((randomized controlled trial[Publication Type] OR randomized[Title/Abstract] OR placebo[Title/Abstract])))
