# Supplementary material for: The effectiveness of vasodilators on chronic obstructive pulmonary disease: A systematic review and meta-analysis
Source: Medicine (Baltimore). 2024 Nov 15;103(46):e39794. doi: 10.1097/MD.0000000000039794 (PMC11576023; doi:10.1097/MD.0000000000039794)
Supplement: Supplementary file 2 [file medi-103-e39794-s002.pdf]

SDC Figure 1

	Vasodilators			Control				Mean Difference	Mean Difference
**Study or Subgroup**	**Mean**	**SD**	**Total**	**Mean**	**SD**	**Total**	**Weight**	**IV, Fixed, 95% CI**	**IV, Fixed, 95% CI**
**13.1.1 ACEIs**									
Curtis 2016	10.4	1.62	31	10.42	1.56	34	5.3%	-0.02 [-0.79, 0.75]	
Di Marco 2010	9.9	0.7	18	9.9	0.7	18	15.3%	0.00 [-0.46, 0.46]	
Oliver 1989	8.34	2.34	9	8.08	1.95	9	0.8%	0.26 [-1.73, 2.25]	
Shrikrishna 2014	9.6	1.7	31	9.5	1.8	36	4.5%	0.10 [-0.74, 0.94]	
**Subtotal (95% CI)**			**89**			**97**	**25.9%**	**0.02 [-0.33, 0.37]**	
Heterogeneity: Chi² = 0.11, df = 3 (P = 0.99); I² = 0%									
Test for overall effect: Z = 0.12 (P = 0.90)									
**13.1.2 ARBs**									
Andreas 2006	8.9	1.6	26	8.3	1.6	24	4.0%	0.60 [-0.29, 1.49]	
**Subtotal (95% CI)**			**26**			**24**	**4.0%**	**0.60 [-0.29, 1.49]**	
Heterogeneity: Not applicable									
Test for overall effect: Z = 1.32 (P = 0.19)									
**13.1.3 CCBs**									
Cheng 1997	9.41	0.93	102	9.65	1.19	100	36.7%	-0.24 [-0.53, 0.05]	
Dujić 1991	5.45	0.55	17	5.6	0.6	15	19.9%	-0.15 [-0.55, 0.25]	
Lee 2004	10.07	2.24	11	11.32	1.85	10	1.0%	-1.25 [-3.00, 0.50]	
**Subtotal (95% CI)**			**130**			**125**	**57.6%**	**-0.23 [-0.46, 0.01]**	
Heterogeneity: Chi² = 1.46, df = 2 (P = 0.48); I² = 0%									
Test for overall effect: Z = 1.89 (P = 0.06)									
**13.1.4 PDE-5 Inhibitors**									
Lederer 2012	9.73	1.21	10	10.27	1.21	10	2.8%	-0.54 [-1.60, 0.52]	
Patrizio 2017	9.21	1.84	18	9.36	1.68	10	1.8%	-0.15 [-1.49, 1.19]	
**Subtotal (95% CI)**			**28**			**20**	**4.6%**	**-0.39 [-1.22, 0.44]**	
Heterogeneity: Chi² = 0.20, df = 1 (P = 0.66); I² = 0%									
Test for overall effect: Z = 0.92 (P = 0.36)									
**13.1.5 NO**									
Tuyet 2022	10.4	1.9	15	9.7	0.4	15	3.3%	0.70 [-0.28, 1.68]	
Vonbank 2002	10.1	1.6	20	10.6	1.9	20	2.7%	-0.50 [-1.59, 0.59]	
**Subtotal (95% CI)**			**35**			**35**	**6.0%**	**0.16 [-0.57, 0.89]**	
Heterogeneity: Chi² = 2.57, df = 1 (P = 0.11); I² = 61%									
Test for overall effect: Z = 0.43 (P = 0.66)									
**13.1.6 Prostacyclins**									
Boeck 2012	7.33	1.55	16	7.84	2.2	16	1.8%	-0.51 [-1.83, 0.81]	
**Subtotal (95% CI)**			**16**			**16**	**1.8%**	**-0.51 [-1.83, 0.81]**	
Heterogeneity: Not applicable									
Test for overall effect: Z = 0.76 (P = 0.45)									
**Total (95% CI)**			**324**			**317**	**100.0%**	**-0.12 [-0.30, 0.06]**	
Heterogeneity: Chi² = 9.60, df = 12 (P = 0.65); I² = 0%									
Test for overall effect: Z = 1.30 (P = 0.19)									
Test for subgroup differences: Chi² = 5.26, df = 5 (P = 0.38), I² = 5.0%									

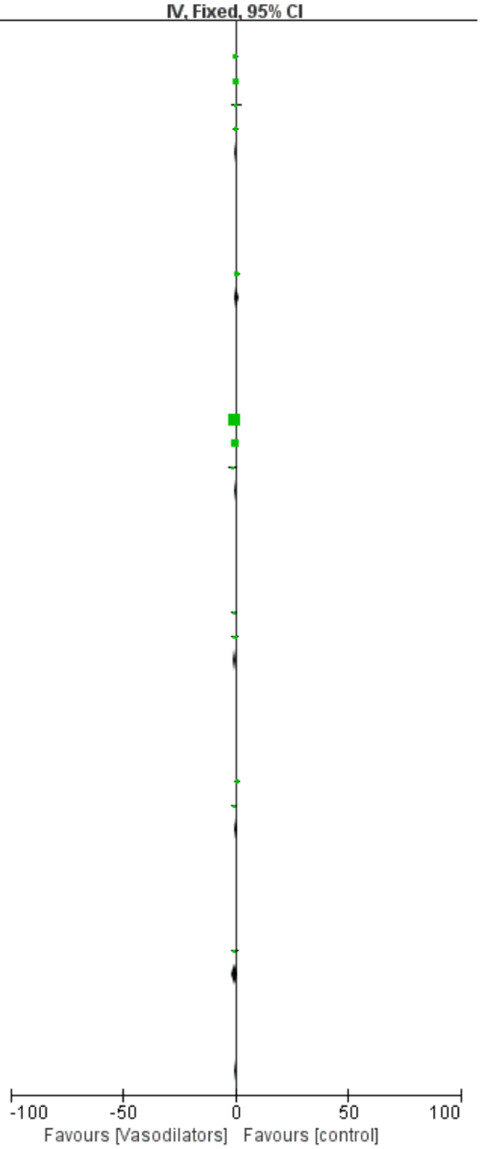

SDC Figure 2

Study or Subgroup	Experimental Mean	Experimental SD	Experimental Total	Control Mean	Control SD	Control Total	Weight	Mean Difference IV, Fixed, 95% CI
**18.1.1 COPD**								
Andreas 2006	8.9	1.6	26	8.3	1.6	24	4.0%	0.60 [-0.29, 1.49]
Boeck 2012	7.33	1.55	16	7.84	2.2	16	1.8%	-0.51 [-1.83, 0.81]
Cheng 1997	9.41	0.93	102	9.65	1.19	100	36.7%	-0.24 [-0.53, 0.05]
Curtis 2016	10.4	1.62	31	10.42	1.56	34	5.3%	-0.02 [-0.79, 0.75]
Di Marco 2010	9.9	0.7	18	9.9	0.7	18	15.3%	0.00 [-0.46, 0.46]
Lederer 2012	9.73	1.21	10	10.27	1.21	10	2.8%	-0.54 [-1.60, 0.52]
Lee 2004	10.07	2.24	11	11.32	1.85	10	1.0%	-1.25 [-3.00, 0.50]
Oliver 1989	8.34	2.34	9	8.08	1.95	9	0.8%	0.26 [-1.73, 2.25]
Shrikrishna 2014	9.6	1.7	31	9.5	1.8	36	4.5%	0.10 [-0.74, 0.94]
Vonbank 2002	10.1	1.6	20	10.6	1.9	20	2.7%	-0.50 [-1.59, 0.59]
**Subtotal (95% CI)**			**274**			**277**	**75.1%**	**-0.15 [-0.35, 0.06]**
Heterogeneity: Chi² = 6.84, df = 9 (P = 0.65); I² = 0%								
Test for overall effect: Z = 1.38 (P = 0.17)								
**18.1.2 COPD-PH**								
Dujić 1991	5.45	0.55	17	5.6	0.6	15	19.9%	-0.15 [-0.55, 0.25]
Patrizio 2017	9.21	1.84	18	9.36	1.68	10	1.8%	-0.15 [-1.49, 1.19]
Tuyet 2022	10.4	1.9	15	9.7	0.4	15	3.3%	0.70 [-0.28, 1.68]
**Subtotal (95% CI)**			**50**			**40**	**24.9%**	**-0.04 [-0.40, 0.32]**
Heterogeneity: Chi² = 2.49, df = 2 (P = 0.29); I² = 20%								
Test for overall effect: Z = 0.20 (P = 0.84)								
**Total (95% CI)**			**324**			**317**	**100.0%**	**-0.12 [-0.30, 0.06]**
Heterogeneity: Chi² = 9.60, df = 12 (P = 0.65); I² = 0%								
Test for overall effect: Z = 1.30 (P = 0.19)								
Test for subgroup differences: Chi² = 0.26, df = 1 (P = 0.61), I² = 0%								

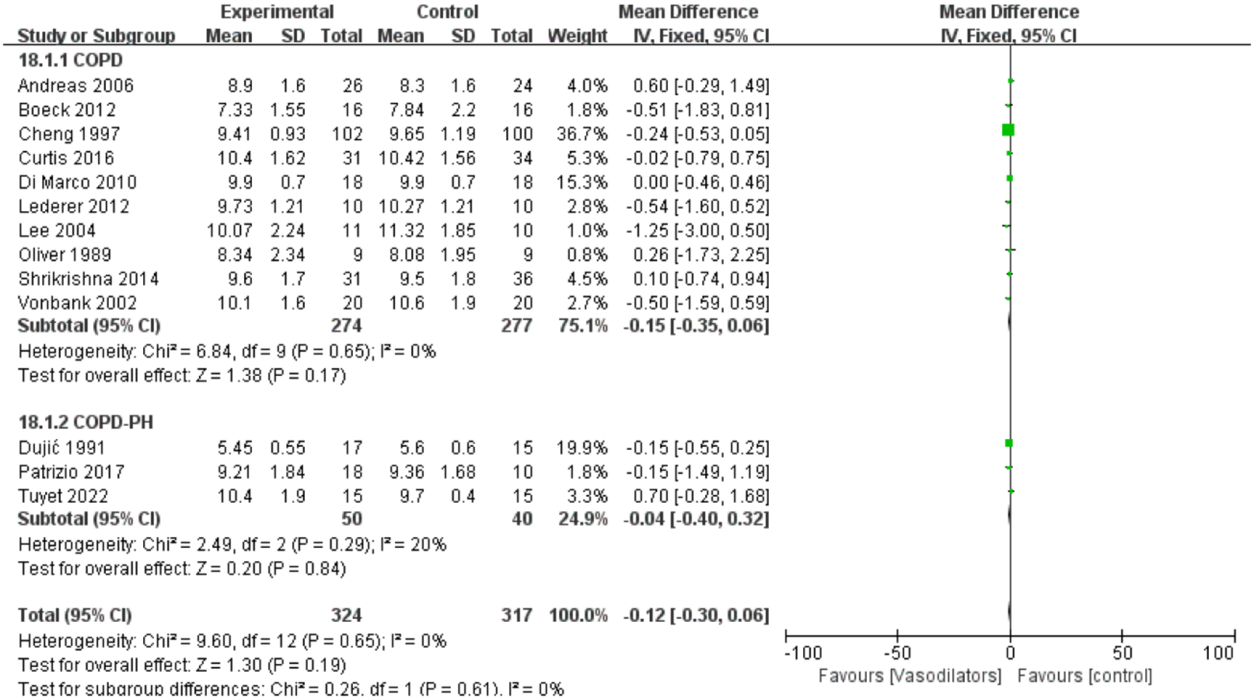

SDC Figure 3

	vasodilators			Control				Mean Difference
Study or Subgroup	Mean	SD	Total	Mean	SD	Total	Weight	IV, Random, 95% CI
**20.1.1 CCBs**								
Cheng 1997	93.8	1.94	102	93.18	1.58	100	43.6%	0.62 [0.13, 1.11]
Lee 2004	93.9	5.6	11	96.2	1.6	10	18.4%	-2.30 [-5.75, 1.15]
**Subtotal (95% CI)**			**113**			**110**	**62.1%**	**-0.32 [-2.99, 2.35]**
Heterogeneity: Tau² = 2.68; Chi² = 2.69, df = 1 (P = 0.10); I² = 63%								
Test for overall effect: Z = 0.23 (P = 0.82)								
**20.1.2 NO**								
Tuyet 2022	94.9	1.4	15	92.2	2	15	37.9%	2.70 [1.46, 3.94]
**Subtotal (95% CI)**			**15**			**15**	**37.9%**	**2.70 [1.46, 3.94]**
Heterogeneity: Not applicable								
Test for overall effect: Z = 4.28 (P < 0.0001)								
**Total (95% CI)**			**128**			**125**	**100.0%**	**0.87 [-1.06, 2.80]**
Heterogeneity: Tau² = 2.17; Chi² = 12.66, df = 2 (P = 0.002); I² = 84%								
Test for overall effect: Z = 0.88 (P = 0.38)								
Test for subgroup differences: Chi² = 4.04, df = 1 (P = 0.04), I² = 75.2%								

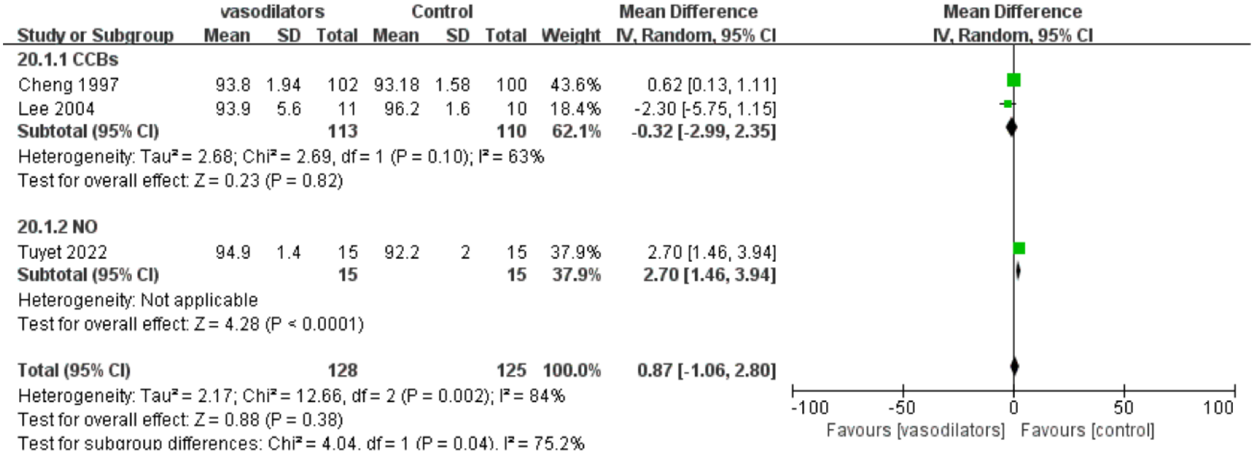

SDC Figure 4

Study or Subgroup	vasodilators Mean	SD	Total	Control Mean	SD	Total	Weight	Mean Difference IV, Random, 95% CI
**19.1.1 COPD**								
Cheng 1997	93.8	1.94	102	93.18	1.58	100	43.6%	0.62 [0.13, 1.11]
Lee 2004	93.9	5.6	11	96.2	1.6	10	18.4%	-2.30 [-5.75, 1.15]
**Subtotal (95% CI)**			**113**			**110**	**62.1%**	**-0.32 [-2.99, 2.35]**
Heterogeneity: Tau² = 2.68; Chi² = 2.69, df = 1 (P = 0.10); I² = 63%								
Test for overall effect: Z = 0.23 (P = 0.82)								
**19.1.2 COPD-PH**								
Tuyet 2022	94.9	1.4	15	92.2	2	15	37.9%	2.70 [1.46, 3.94]
**Subtotal (95% CI)**			**15**			**15**	**37.9%**	**2.70 [1.46, 3.94]**
Heterogeneity: Not applicable								
Test for overall effect: Z = 4.28 (P < 0.0001)								
**Total (95% CI)**			**128**			**125**	**100.0%**	**0.87 [-1.06, 2.80]**
Heterogeneity: Tau² = 2.17; Chi² = 12.66, df = 2 (P = 0.002); I² = 84%								
Test for overall effect: Z = 0.88 (P = 0.38)								
Test for subgroup differences: Chi² = 4.04, df = 1 (P = 0.04), I² = 75.2%								

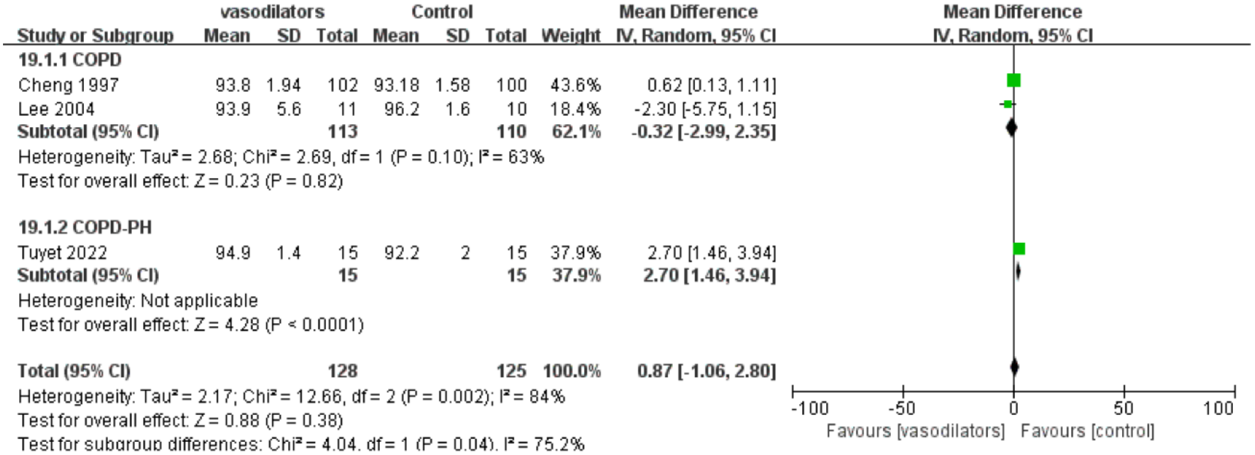

SDC Figure 5

Study or Subgroup	Vasodilators Mean	Vasodilators SD	Vasodilators Total	Control Mean	Control SD	Control Total	Weight	Mean Difference IV, Random, 95% CI
Andreas 2006	0.87	0.3	26	0.95	0.6	24	9.4%	-0.08 [-0.35, 0.19]
Andrew 2014	1.05	0.38	56	0.99	0.4	57	12.6%	0.06 [-0.08, 0.20]
Bozorgmehr 2020	1.9	0.4	20	1.09	0.6	20	8.2%	0.81 [0.49, 1.13]
Cheng 1997	0.92	0.42	102	0.96	0.53	100	12.9%	-0.04 [-0.17, 0.09]
Curtis 2016	1.09	0.72	31	1.29	0.71	34	7.5%	-0.20 [-0.55, 0.15]
Di Marco 2010	1.3	0.36	18	1.32	0.34	18	10.4%	-0.02 [-0.25, 0.21]
Lederer 2012	1.1	0.47	10	1.1	0.38	10	7.0%	0.00 [-0.37, 0.37]
Lee 2004	1	0.34	11	0.95	0.34	10	8.8%	0.05 [-0.24, 0.34]
Robert 2008	1.48	0.68	23	1.36	0.59	20	6.9%	0.12 [-0.26, 0.50]
Stolz 2008	1.07	0.4	15	1.28	0.7	17	6.7%	-0.21 [-0.60, 0.18]
Vonbank 2002	0.86	0.26	20	1.07	0.38	10	9.5%	-0.21 [-0.47, 0.05]
**Total (95% CI)**			**332**			**320**	**100.0%**	**0.02 [-0.11, 0.16]**

Heterogeneity: $Tau^2 = 0.03$; $Chi^2 = 31.87$, df = 10 (P = 0.0004); $I^2 = 69\%$
Test for overall effect: Z = 0.33 (P = 0.74)

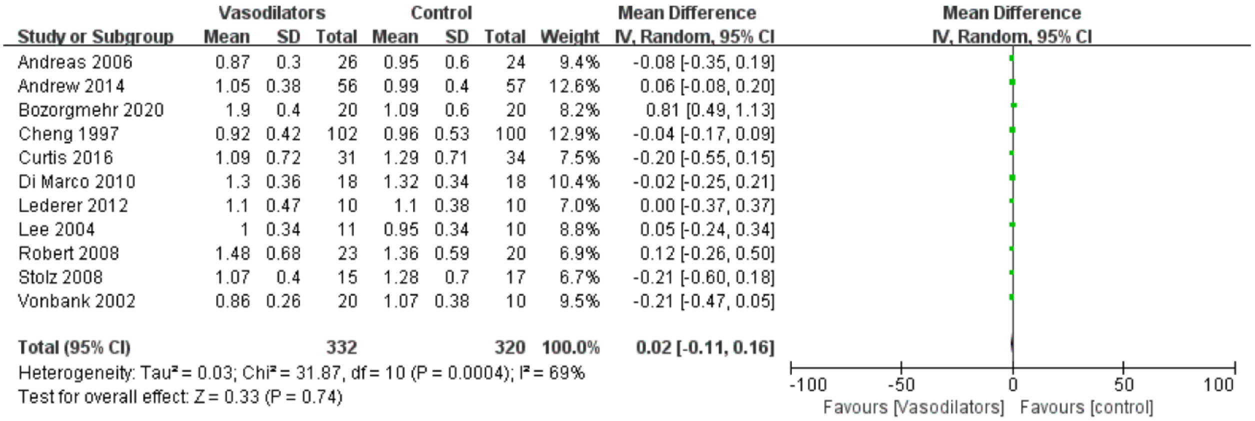

SDC Figure 6

Study or Subgroup	Vasodilators Mean	Vasodilators SD	Vasodilators Total	Control Mean	Control SD	Control Total	Weight	Mean Difference IV, Random, 95% CI
Andreas 2006	0.87	0.3	26	0.95	0.6	24	7.3%	-0.08 [-0.35, 0.19]
Andrew 2014	1.05	0.38	56	0.99	0.4	57	24.9%	0.06 [-0.08, 0.20]
Bozorgmehr 2020	1.9	0.4	20	1.09	0.6	20	0.0%	0.81 [0.49, 1.13]
Cheng 1997	0.92	0.42	102	0.96	0.53	100	29.5%	-0.04 [-0.17, 0.09]
Curtis 2016	1.09	0.72	31	1.29	0.71	34	4.2%	-0.20 [-0.55, 0.15]
Di Marco 2010	1.3	0.36	18	1.32	0.34	18	9.8%	-0.02 [-0.25, 0.21]
Lederer 2012	1.1	0.47	10	1.1	0.38	10	3.7%	0.00 [-0.37, 0.37]
Lee 2004	1	0.34	11	0.95	0.34	10	6.1%	0.05 [-0.24, 0.34]
Robert 2008	1.48	0.68	23	1.36	0.59	20	3.6%	0.12 [-0.26, 0.50]
Stolz 2008	1.07	0.4	15	1.28	0.7	17	3.4%	-0.21 [-0.60, 0.18]
Vonbank 2002	0.86	0.26	20	1.07	0.38	10	7.5%	-0.21 [-0.47, 0.05]
**Total (95% CI)**			**312**			**300**	**100.0%**	**-0.03 [-0.10, 0.04]**

Heterogeneity: $Tau^2 = 0.00$; $Chi^2 = 6.14$, $df = 9$ ($P = 0.73$); $I^2 = 0\%$
Test for overall effect: $Z = 0.79$ ($P = 0.43$)

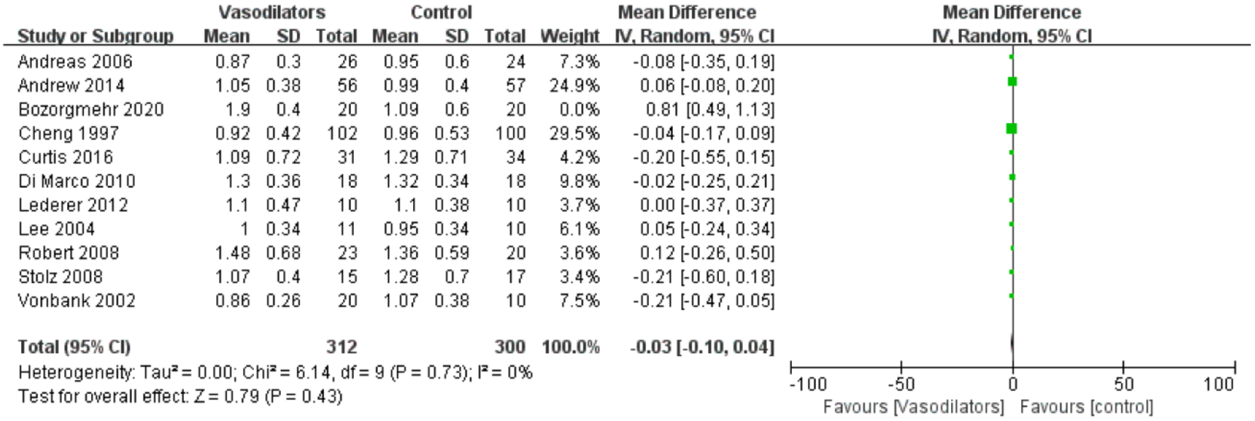

SDC Figure 7

	Vasodilators			Control				Mean Difference
**Study or Subgroup**	**Mean**	**SD**	**Total**	**Mean**	**SD**	**Total**	**Weight**	**IV, Fixed, 95% CI**
Andrew 2014	40.1	15.4	56	38.9	15.2	57	12.3%	1.20 [-4.44, 6.84]
Bozorgmehr 2020	45.4	13.4	20	41.5	19.1	20	3.7%	3.90 [-6.33, 14.13]
Cheng 1997	46.22	8.68	102	46.84	9.86	100	59.4%	-0.62 [-3.18, 1.94]
Curtis 2016	48.1	29.55	31	51.62	27.18	34	2.0%	-3.52 [-17.36, 10.32]
Dujić 1991	53.1	14.68	17	52.3	15.7	17	3.7%	0.80 [-9.42, 11.02]
Lee 2004	40	11.6	11	36	10.5	10	4.4%	4.00 [-5.45, 13.45]
Oliver 1989	32.9	8.4	9	32.1	9.3	9	5.8%	0.80 [-7.39, 8.99]
Patrizio 2017	54.6	22.7	18	45.63	22.48	10	1.3%	8.97 [-8.47, 26.41]
Shrikrishna 2014	46.1	21.71	31	42.7	22.2	36	3.5%	3.40 [-7.14, 13.94]
Stolz 2008	35	12	20	42	14	10	3.8%	-7.00 [-17.15, 3.15]
**Total (95% CI)**			**315**			**303**	**100.0%**	**0.07 [-1.90, 2.05]**

Heterogeneity: Chi² = 5.19, df = 9 (P = 0.82); I² = 0%
Test for overall effect: Z = 0.07 (P = 0.94)

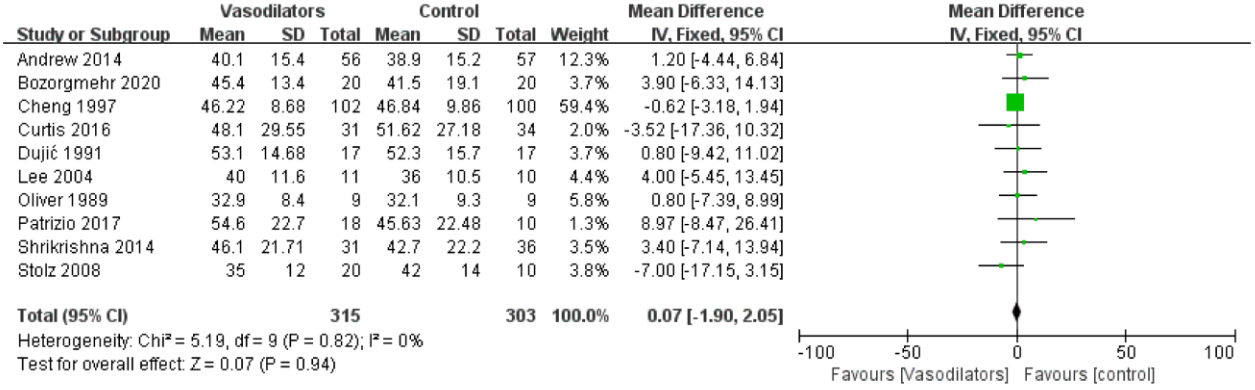

SDC Figure 8

Study or Subgroup	Vasodilators Mean	SD	Total	Control Mean	SD	Total	Weight	Mean Difference IV, Random, 95% CI
Bozorgmehr 2020	45.4	13.4	20	41.5	19.1	20	21.0%	3.90 [-6.33, 14.13]
Lederer 2012	39	11.54	10	40	9.35	10	25.8%	-1.00 [-10.21, 8.21]
Lee 2004	40	11.6	11	36	10.5	10	24.5%	4.00 [-5.45, 13.45]
Patrizio 2017	54.6	22.7	18	45.63	22.48	10	7.3%	8.97 [-8.47, 26.41]
Stolz 2008	35	12	20	42	14	10	21.3%	-7.00 [-17.15, 3.15]
**Total (95% CI)**			**79**			**60**	**100.0%**	**0.70 [-4.02, 5.42]**

Heterogeneity: Tau² = 0.38; Chi² = 4.05, df = 4 (P = 0.40); I² = 1%

Test for overall effect: Z = 0.29 (P = 0.77)

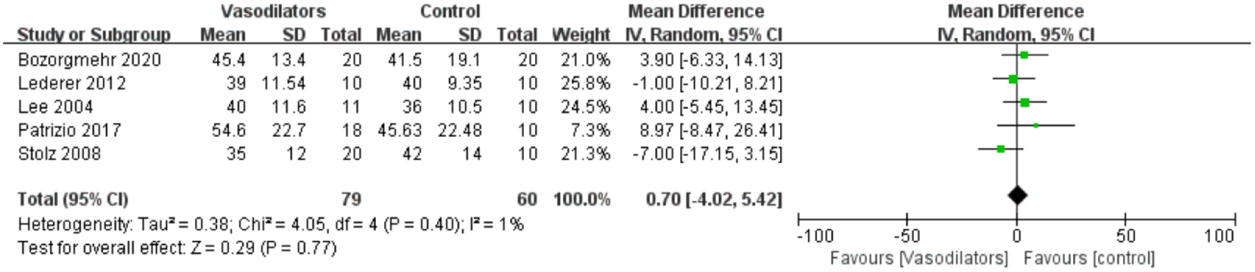

SDC Figure 9

Study or Subgroup	Vasodilators Mean	SD	Total	Control Mean	SD	Total	Weight	Mean Difference IV, Random, 95% CI
Andreas 2006	37.8	7.5	16	38.5	8.5	16	16.0%	-0.70 [-6.25, 4.85]
Boeck 2012	32.8	9.4	18	34.4	8.5	18	14.4%	-1.60 [-7.45, 4.25]
Di Marco 2010	36.6	3.5	15	36.4	4.3	15	62.7%	0.20 [-2.61, 3.01]
Phillips 2021	24.1	14.3	26	23.4	16.1	24	6.9%	0.70 [-7.77, 9.17]
**Total (95% CI)**			**75**			**73**	**100.0%**	**-0.17 [-2.39, 2.05]**

Heterogeneity: $Tau^2 = 0.00$; $Chi^2 = 0.37$, df = 3 (P = 0.95); $I^2 = 0\%$

Test for overall effect: Z = 0.15 (P = 0.88)

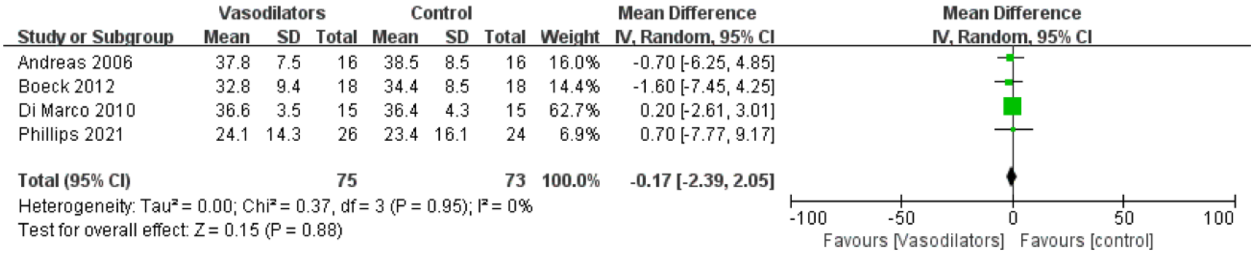

SDC Figure 10

A Funnel plot

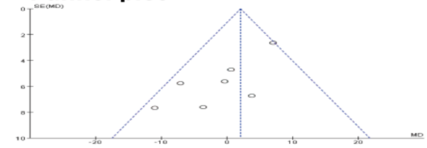

Egger's test

Std_Eff	Coefficient	Std. err.	t	P>|t|	[95% conf.	interval]
slope	.8737242	.3570219	2.45	0.058	-.0440298	1.791478
bias	-2.881265	1.249795	-2.31	0.069	-6.093966	.3314354

Begg's test

```
Kendall's score =     -9.00
    SE of score =     6.658
              z =     -1.50
     Prob > |z| =    0.2296
```

B Funnel plot

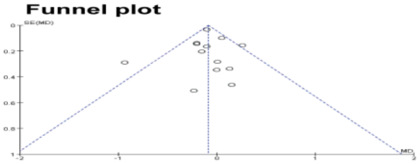

Egger's test

Std_Eff	Coefficient	Std. err.	t	P>|t|	[95% conf.	interval]
slope	.3454837	.2627661	1.31	0.215	-.2328607	.923828
bias	-1.657461	.9115611	-1.82	0.096	-3.663794	.3488713

Begg's test

```
Kendall's score =    -20.00
    SE of score =    16.391
              z =     -1.28
     Prob > |z| =    0.2464
```

C Funnel plot

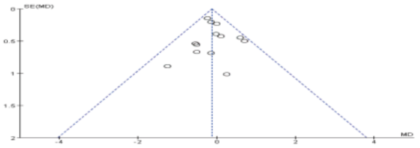

Egger's test

Std_Eff	Coefficient	Std. err.	t	P>|t|	[95% conf.	interval]
slope	-.1631318	.2049417	-0.80	0.443	-.6142055	.2879418
bias	.2682641	.7145293	0.38	0.714	-1.304404	1.840932

Begg's test

```
Kendall's score =     -8.00
    SE of score =    16.391
              z =     -0.55
     Prob > |z| =    0.6693
```

D Funnel plot

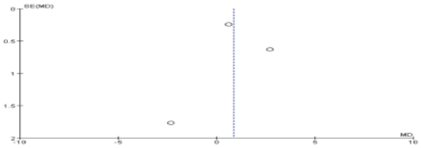

Egger's test

Std_Eff	Coefficient	Std. err.	t	P>|t|	[95% conf.	interval]
slope	.27977	.8959591	0.31	0.807	-11.10447	11.66401
bias	.5650873	4.020381	0.14	0.911	-50.5187	51.64887

Begg's test

```
Kendall's score =     -1.00
    SE of score =     1.915
              z =     -1.04
     Prob > |z| =    1.0000
```

E Funnel plot

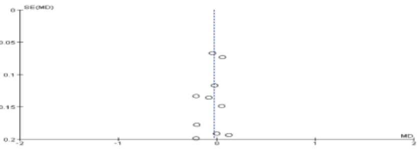

Egger's test

Std_Eff	Coefficient	Std. err.	t	P>|t|	[95% conf.	interval]
slope	-.1196378	.3613278	-0.33	0.748	-.937018	.6977424
bias	.5014649	1.372072	0.37	0.723	-2.602377	3.605307

Begg's test

```
Kendall's score =      3.00
    SE of score =    12.845
              z =      0.16
     Prob > |z| =    0.8763
```

F Funnel plot

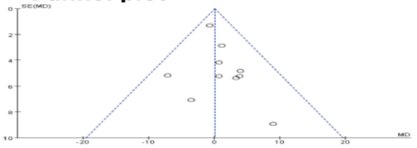

Egger's test

Std_Eff	Coefficient	Std. err.	t	P>|t|	[95% conf.	interval]
slope	-.1005845	.1631614	-0.62	0.555	-.4768354	.2756665
bias	.5198404	.6367167	0.82	0.438	-.9484308	1.988112

Begg's test

```
Kendall's score =     11.00
    SE of score =    11.180
              z =      0.89
     Prob > |z| =    0.3711
```

G Funnel plot

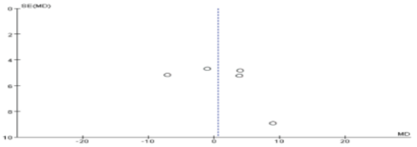

Egger's test

Std_Eff	Coefficient	Std. err.	t	P>|t|	[95% conf.	interval]
slope	.646797	.8216037	0.79	0.489	-1.967913	3.261507
bias	-2.173683	2.1107	-1.03	0.379	-8.890873	4.543507

Begg's test

```
Kendall's score =      0.00
    SE of score =     4.082
              z =     -0.24
     Prob > |z| =    1.0000
```

H Funnel plot

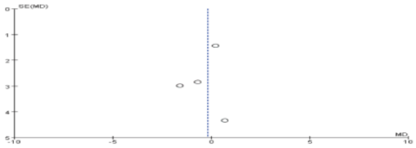

Egger's test

Std_Eff	Coefficient	Std. err.	t	P>|t|	[95% conf.	interval]
slope	.2818922	.6338724	0.44	0.700	-2.445441	3.009225
bias	-.9718224	1.925971	-0.50	0.664	-9.258607	7.314963

Begg's test

```
Kendall's score =      0.00
    SE of score =     2.944
              z =     -0.34
     Prob > |z| =    1.0000
```

I Funnel plot

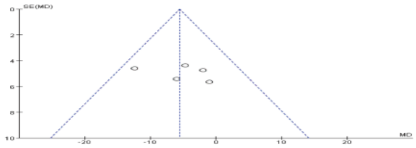

Egger's test

Std_Eff	Coefficient	Std. err.	t	P>|t|	[95% conf.	interval]
slope	1.171836	.143257	8.18	0.004	.7159278	1.627743
bias	-5.036779	.4954403	-10.17	0.002	-6.613491	-3.460067

Begg's test

```
Kendall's score =     -8.00
    SE of score =     4.082
              z =     -2.20
     Prob > |z| =    0.0864
```
